# The type of blood used to feed *Aedes aegypti* females affects their cuticular and internal free fatty acid (FFA) profiles

**DOI:** 10.1371/journal.pone.0251100

**Published:** 2021-04-30

**Authors:** Agata Kaczmarek, Anna Katarzyna Wrońska, Mieczysława Irena Boguś, Michalina Kazek, Aleksandra Gliniewicz, Ewa Mikulak, Marta Matławska

**Affiliations:** 1 Witold Stefański Institute of Parasitology, Polish Academy of Sciences, Warsaw, Poland; 2 BIOMIBO, Warsaw, Poland; 3 National Institute of Public Health—National Institute of Hygiene, Warsaw, Poland; Fundacao Oswaldo Cruz Instituto Rene Rachou, BRAZIL

## Abstract

*Aedes aegypti*, the primary vector of various arthropod-borne viral (arboviral) diseases such as dengue and Zika, is a popular laboratory model in vector biology. However, its maintenance in laboratory conditions is difficult, mostly because the females require blood meals to complete oogenesis, which is often provided as sheep blood. The outermost layer of the mosquito cuticle is consists of lipids which protects against numerous entomopathogens, prevents desiccation and plays an essential role in signalling processes. The aim of this work was to determine how the replacement of human blood with sheep blood affects the cuticular and internal FFA profiles of mosquitoes reared in laboratory culture. The individual FFAs present in cuticular and internal extracts from mosquito were identified and quantified by GC–MS method. The normality of their distribution was checked using the Kolmogorov-Smirnov test and the Student’s t-test was used to compare them. GC-MS analysis revealed similar numbers of internal and cuticular FFAs in the female mosquitoes fed sheep blood by membrane (MFSB) and naturally fed human blood (NFHB), however MFSB group demonstrated 3.1 times greater FFA concentrations in the cuticular fraction and 1.4 times the internal fraction than the NFHB group. In the MFSB group, FFA concentration was 1.6 times higher in the cuticular than the internal fraction, while for NFHB, FFA concentration was 1.3 times lower in the cuticular than the internal fraction. The concentration of C18:3 acid was 223 times higher in the internal fraction than the cuticle in the MHSB group but was absent in the NFHB group. MFSB mosquito demonstrate different FFA profiles to wild mosquitoes, which might influence their fertility and the results of vital processes studied under laboratory conditions. The membrane method of feeding mosquitoes is popular, but our research indicates significant differences in the FFA profiles of MFSB and NFHB. Such changes in FFA profile might influence female fertility, as well as other vital processes studied in laboratory conditions, such as the response to pesticides. Our work indicates that sheep blood has potential shortcomings as a substitute feed for human blood, as its use in laboratory studies may yield different results to those demonstrated by free-living mosquitoes.

## Introduction

Mosquitoes are well known to be important vectors of pathogens with serious effects on human health. Geographically, tropical and subtropical areas are the most suitable regions for optimal growth and reproduction of mosquitoes, and hence mosquito-borne diseases pose a great risk to people in this part of the world. However, climate change, and the increase in human travel and migration observed in recent years has resulted in the spread of these diseases to areas where they had previously been non-existent [1–4]. *Aedes aegypti* is a major public health concern due to its ability to be efficient vectors of malaria, dengue, Chikungunya, Zika, and other arboviruses [5–13].

The rearing of mosquitoes is important for both the theoretical and applied sciences. A large amount of research has been conducted on mosquitos and mosquito-borne diseases, and such procedures demand a constant supply of laboratory-reared insects. Some experiments, such as monitoring insecticide resistance, production of mosquito pathogens for vaccine development or improving methods of controlling insect populations, require a fast method of expanding laboratory populations [14–18].

However, the maintenance of mosquitoes in laboratory conditions is a challenging task, with the most important duty being ensuring the availability of blood meal, as this facilitates two crucial processes in female mosquitoes: oogenesis and egg production. *Ae*. *aegyptii* are mostly anthropophilic and of all the host species (human, dog, cat, rodent, bovine, porcine, and avian) available to *Ae*. *aegypti* in its natural rural and urban habitats, humans are often the most abundant and consistently available [19]. This anthropophagous feeding pattern is associated with two behaviours that are unusual for females of most mosquito species: imbibing blood multiple times during each egg laying cycle and infrequent feeding on plant sugars. The biochemical characteristics of human blood also provide a fitness advantage compared with blood from other vertebrate species [19, 20]. For example, *Ae*. *aegypti* ingesting low-isoleucine human blood accumulate more energy reserves and gain a fitness advantage over those fed on high-isoleucine rodent or chicken blood. Also females fed human blood demonstrated a greater accumulation of triglycerides (lipids) compared with those fed chicken or mouse blood [19]. Research in Thailand suggests that *Ae*. *aegypti* prefers to feed on human blood as a single host species, and multiple-host bloodmeals included at least one human host; although blood from some other hosts, including bovines, swine, cats, rats, and chickens were detected, they represented less than 1% of bloodmeals [21].

Currently, in laboratory conditions, blood supplies are provided by live animals, human volunteers and whole blood via a artificial membrane feeding techniques (glass plate, metal plate, Hemotek membrane feeding method) [22–24]. However, these approaches all pose a challenge: they are costly, require a special permit, need a trained team to perform them and the blood must be pathogen-free and safe for both the laboratory staff and the reared mosquito. Moreover, different mosquito species prefer certain vertebrate blood sources over others [25]. In laboratory conditions, a popular replacement for human blood in mosquito rearing is sheep blood, and research has shown that feeding with defibrinated sheep blood is more effective than washed sheep erythrocytes [26], The major blood cell protein, haemoglobin, stimulated yolk deposition in mosquito when it was obtained from pigs, but not from humans, cows or sheep [27]. In addition, membrane feeding on sheep blood resulted in lower survival, fecundity and hatching rates of *Ae*. *aegypti* compared with feeding by human blood [28], and rabbit blood, especially citrated blood, enables more efficient maintenance of laboratory colonies of Culicidae [29]. However, no significant difference of fecundity, oviposition rate or fertility was observed between mosquitoes fed with cattle or human blood, when using artificial membrane feeding techniques [24].

Additionally, the nutritional deficits of a low-quality host blood meal can be offset by using mixed blood, such as chicken and rabbit blood, which may enhance the diversity of gut microbiota in *Ae*. *aegypti* [30]. Therefore the nutritional composition of blood from vertebrate animal sources is a crucial factor in mosquito rearing.

Host preference is a complex phenomenon and has implications for the development of artificial blood meal replacement diets [23, 29, 31]. It is well known that egg production and vitellogenesis require protein, and differences in blood meal composition can result in lower levels of egg production [23]. However, there is a lack of information on how the replacement of human blood in laboratory conditions affects the lipid profile of mosquitoes, particularly its free fatty acid (FFA) content.

Lipids are important components of cells and a significant factor in the well-being of mosquitoes. The lipids of insect cuticle are a diverse group of compounds which vary significantly in their composition. The diversity of lipids is dependent on diet and climate condition. Cuticular lipids, including FFAs, perform many important functions and help maintain the homeostasis of the insect body; most importantly, they minimize transpiration from the insect and protect it from desiccation [32, 33]. Cuticular lipids are also involved in several biochemical, physiological, and semiochemical (behaviour and signalling) processes [33–37]. The composition of cuticular lipids is also a crucial factor in the susceptibility or resistance of various insect species to fungal invasion [38–41]. Cuticular fatty acids are toxic and fungistatic but also may be stimulatory to fungal growth; for example, palmitoleic acid enhances mycelial growth, but is toxic to the conidia of *Erynia variabilis* [42]. The toxic effects of palmitoleic acid can be mitigated by the presence of a sufficient concentration of oleic acid [43].

Internal lipids represent integral and essential parts of the cell membrane and an important source of energy. The greatest concentration of internal lipids is found in the fat body and hemolymph. The fat body is considered as functionally analogous to the vertebrate liver. Its main function is the storage of carbohydrates, lipids and proteins, as well as the synthesis of lipids, trehalose (hemolymph sugar) and the yolk, which is stored in developing eggs [44]. Lipids are also important for mosquito reproduction process as a source of energy for the developing embryo [45]. In the female mosquito, the lipid metabolism is connected with the gonadotrophic cycle, and the levels of FFAs such as C16:0 and C18:1 act as two of the regulating factors of lipid metabolism [46].

Lipids also play an essential role in mosquitoes as factors of resistance or susceptibility to viruses. Lipid droplets (LDs) have been found to accumulate in *Ae*. *aegypti* Aag2 cell lines infected by dengue virus (DENV) [47]. LDs, constituted by fatty acids, among others, occur in the fat body of mosquitoes, where they maintain homeostasis by regulating the lipid metabolism [48]. Accumulation of LDs during DENV infection has been found to play a paradoxical role [49, 50]. On one hand, changes in lipid environment may serve as part of an antiviral strategy, as activation of an immune signalling pathways, like the Toll and the Imd, during infection increases the content of LDs in the mosquito midgut. However, these accumulated lipids could also be used as a source of energy by the invading DENV [47].

The aim of this work was to determine whether the method of feeding mosquitoes with sheep blood, commonly used in laboratories, is a good substitute for natural feeding of human blood. A testable hypothesis was to check whether both the type of blood and feeding method (natural feeding with human blood vs. membrane feeding with sheep blood) could affect mosquito fatty acid profiles.

## Results

The present work analysed the chemical composition of cuticular and internal FFAs in MFSB and NFHB mosquitoes. Our findings indicate 1.5—times lower body mass (per insect) in the MFSB mosquitoes than the NFHB group. For each feeding regime, three different extractions were performed: one using petroleum ether (I), one with dichloromethane (II) and another with dichloromethane in combination with sonification. The NFHB mosquitoes yielded 0.01 mg of cuticular extracts per insect: 0.002 mg in petroleum ether (I) and 0.008 mg in dichloromethane (II) extract. They also yielded 0.011 mg of internal extracts per insect.

The MFSB females yielded higher amounts of cuticular extracts than the NFHB females, amounting to 0.032 mg per insect: 0.011 mg in extract I and 0.021 mg in extract II; however, lower levels of internal extracts were observed: 0.011 mg per insect in NFHB vs.0.004 mg per insect in MFSB. The masses of the extracts are shown in Table 1.

**Table 1:** The numbers of *AE*. *aegypti* females used in the experiment and masses of extracts.

Extracts made from:	N	Wet mass of whole analysed insects (g)	Extract mass					
			mg			mg/insect		
			I	II	III	I	II	III
**MFSB**	537	0.08	5.94	11.09	2.32	0.011	0.021	0.004
**NFHB**	175	0.04	0.31	1.41	1.84	0.002	0.008	0.011

N–total number of individuals; I–petroleum ether extract; II–dichloromethane extract; III–dichloromethane extract after sonification.

**Fig 1:**
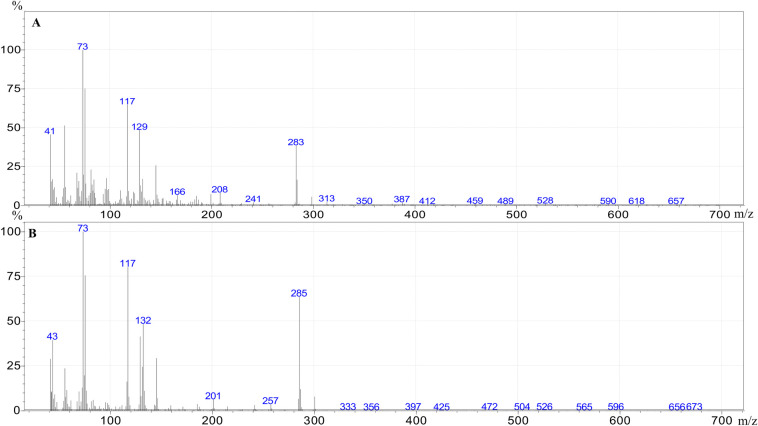
Mass spectra of the trimethylsilyl ester of tetradecenoic acid (A) and tetradecanoic acid (B).

The individual FFAs present in each extract were identified and quantified by GC–MS. Example mass spectra of the trimethylsilyl (TMS) esters of tetradecenoic acid (C14:1) and tetradecanoic acid (C14:0) are given in Fig 1.

A comparison of the FFA profiles of the cuticle surface (sum of I and II extract) and internal structure are shown in Table 2; the raw data, normal distribution and statistical significance are given in S1 Table.

**Table 2:** Fatty acid contents in the cuticular and internal lipids of the female mosquito.

FFA	Cuticle (sum of I and II extract)		Internal (III extract)	
	(μg/g of insect body±SD)		(μg/g of insect body±SD)	
	MFSB	NFHB	MFSB	NFHB
**pentanoic acid C5:0**	2.86±0.51	2.04±0.22	ND*	0.89±0.16*
**hexanoic acid C6:0**	49.78±3.14**	16.68±0.15 **	15.91±0.97*	12.94±1.00*
**heptanoic acid C7:0**	4.91±0.44*	1.61±0.25*	2.41±0.79	1.70±0.34
**octanoic acid C8:0**	11.77±0.46**	4.03±0.22**	6.26±0.22*	4.57±0.44*
**nonanoic acid C9:0**	36.58±1.99**	11.43±0.23**	16.35±1.26*	10.68±0.29*
**decanoic acid C10:0**	4.68±0.47*	1.85±0.27*	3.52±0.24*	1.85±0.21*
**dodecanoic acid C12:0**	57.75±2.83**	16.51±0.85**	38.60±1.33**	24.72±0.79**
**tetradecenoic acid C14:1**	90.30±0.89**	31.25±0.28**	61.01±2.83*	50.78±0.78*
**tetradecanoic acid C14:0**	530.65±9.21**	164.35±1.93**	348.90±7.25**	243.83±6.27**
**pentadecanoic acid C15:0**	9.19±0.99*	3.79±0.35*	6.63±0.53*	3.87±0.65*
**hexadecenoic acid C16:1**	6262.79±142.00**	2 028.14±33.39**	3872.83±24.83**	2 840.37±92.99**
**hexadecanoic acid C16:0**	4324.19±74.10**	1 230.08±12.27**	2654.12±31.04**	1 804.78±14.76**
**heptadecenoic acid C17:1**	19.94±0.76**	3.63±0.43**	10.46±0.87*	5.51±1.85*
**heptadecanoic acid C17:0**	13.12±0.27**	4.29±0.55**	9.74±0.32*	4.83±1.54*
**octadecatrienoic acid C18:3**	2.67±1.14*	ND*	596.74±16.08**	ND**
**octadecadienoic acid C18:2**	1820.23±36.90**	409.49±16.95**	980.78±61.84*	521.53±116.13*
**octadecenoic acid C18:1**	7474.31±238.00**	2856.86±141.36**	4245.24±249.78*	3507.95±240.33*
**octadecanoic acid C18:0**	903.37±34.40**	262.61±9.58**	595.87±17.46**	380.66±7.87**
**eicosapentaenoic acid C20:5**	105.70±1.75**	51.09±3.17**	69.76±1.74	75.33±9.02
**eicosatetraenoic acid C20:4**	114.93±6.26**	36.79±1.80**	87.15±6.70*	51.87±3.70*
**eicosanoic acid C20:0**	47.05±3.30**	13.24±1.83**	25.51±0.96	18.17±3.83
**Sum of FFA**	21904.44±502.49**	7155.17±112.37**	13676.98±215.66**	9577.90±291.88**

FFA- free fatty acids; SD—standard deviation; Extract I- petroleum ether extract; Extract II- dichloromethane extracts, Extract III- dichloromethane extracts after sonification; ND–not detected; MFSB–membrane feeding sheep blood; NFHB–natural feeding human blood; The raw data, normality of distribution and p-value (Student’s t-test) are given in S1 Table.

The MFSB was found to have higher total FFAs content in both the cuticular (21.90±0.50 mg/g of insect body) and internal extracts (13.68±0.22 mg/g of insect body). Although the total FFAs concentration was higher in the cuticular extracts than in the internal extracts in the MFSB group, the opposite was observed in the NFHB group: greater FFAs content was observed in the internal extract (9.58±0.29 mg/g of insect body) than the cuticular extract (7.16±0.11 mg/g of insect body).

**Fig 2:**
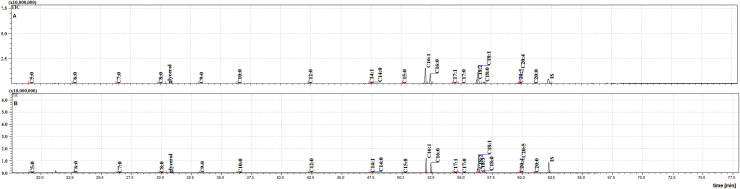
The TIC chromatogram of TMS esters of the ether extract from NFHB (A) and MFSB (B). Internal standard (IS, 19-methylarachidic acid); fatty acids and molecular ions: pentanoic acid (C5:0, m/ z = 174), hexanoic acid (C6:0, m/z = 188), heptanoic acid (C7:0, m/z = 202), octanoic acid (C8:0, m/z = 216), nonanoic acid (C9:0, m/z = 230), decanoic acid (C10:0, m/z = 244), dodecanoic acid (C12:0, m/z = 272), tetradecenoic acid (C14:1, m/z = 298), tetradecanoic acid (C14:0, m/z = 300), pentadecanoic acid (C15:0, m/z = 314), hexadecenoic acid (C16:1, m/z = 326), hexadecanoic acid (C16:0, m/z = 328), heptadecenoic acid (C17:1, m/z = 340), heptadecanoic acid (C17:0, m/z = 342), octadecadienic acid (C18:2, m/z = 352), octadecenoic acid (C18:1, m/z = 354), octadecanoic acid (C18:0, m/z = 356), eicosatetraenoic acid (C20:4, m/z = 376), eicosapentaenoic acid (C20:5, m/z = 374), eicosanoic acid (C20:0, m/z = 384).

**Fig 3:**
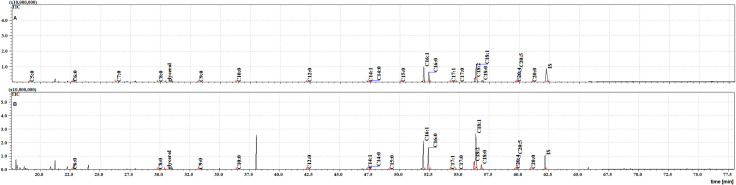
The TIC chromatograms of TMS esters of the dichloromethane extract from NFHB (A) and MFSB (B). Internal standard (IS, 19-methylarachidic acid); fatty acids and molecular ions: pentanoic acid (C5:0, m/ z = 174), hexanoic acid (C6:0, m/z = 188), heptanoic acid (C7:0, m/z = 202), octanoic acid (C8:0, m/z = 216), nonanoic acid (C9:0, m/z = 230), decanoic acid (C10:0, m/z = 244), dodecanoic acid (C12:0, m/z = 272), tetradecenoic acid (C14:1, m/z = 298), tetradecanoic acid (C14:0, m/z = 300), pentadecanoic acid (C15:0, m/z = 314), hexadecenoic acid (C16:1, m/z = 326), hexadecanoic acid (C16:0, m/z = 328), heptadecenoic acid (C17:1, m/z = 340), heptadecanoic acid (C17:0, m/z = 342), octadecatrienoic acid (C18:3, m/z = 350), octadecadienic acid (C18:2, m/z = 352), octadecenoic acid (C18:1, m/z = 354), octadecanoic acid (C18:0, m/z = 356), eicosatetraenoic acid (C20:4, m/z = 376), eicosapentaenoic acid (C20:5, m/z = 374), eicosanoic acid (C20:0, m/z = 384).

**Fig 4:**
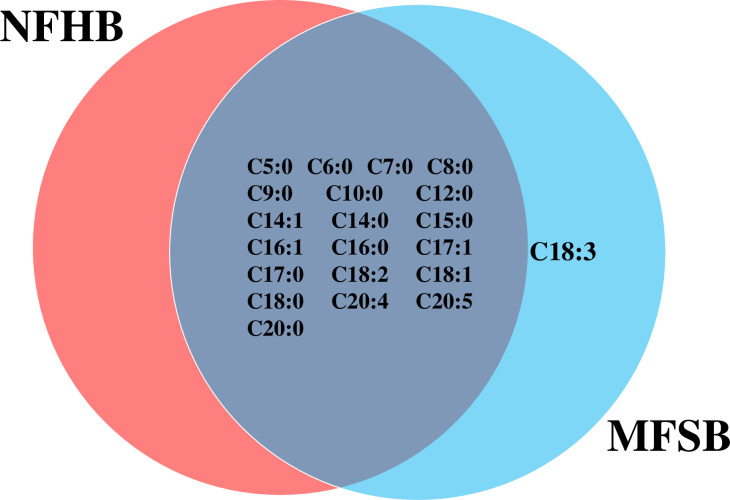
Venn diagram illustrating the differences in cuticular FFAs in NFHB and MFSB mosquitoes.

The two cuticular extracts (petroleum ether—extract I and dichloromethane—extract II) from MFSB mosquitoes contained 21 FFAs from C5:0 to C20:0, thirteen saturated (C5:0, C6:0, C7:0, C8:0, C9:0, C10:0, C12:0, C14:0, C15:0, C16:0, C17:0, C18:0 and C20:0) and eight unsaturated forms (C14:1, C16:1, C17:1, C18:3, C18:2, C18:1, C20:5 and C20:4). The total ion current (TIC) chromatogram of fatty acids (TMS esters) of the ether extract (Extract I) and of dichloromethane extract (Extract II) from MFSB (A) and NFHB (B) are presented in Figs 2 and 3, respectively. The internal extract from MFSB demonstrated the similar content to cuticular, with the exception of C5:0 FFA, which was absent in extract III. The difference in cuticular FFAs in each group is shown in the Venn diagram in Fig 4.

**Fig 5:**
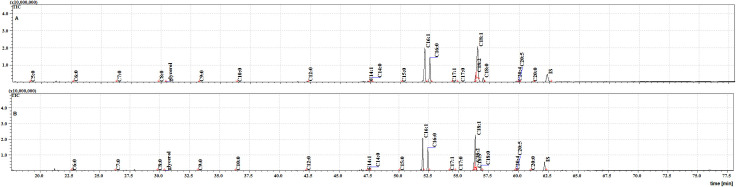
The TIC chromatogram of TMS esters of the dichloromethane extract after sonication from NFHB (A) and MFSB (B). Internal standard (IS, 19-methylarachidic acid); fatty acids and molecular ions: pentanoic acid (C5:0, m/ z = 174), hexanoic acid (C6:0, m/z = 188), heptanoic acid (C7:0, m/z = 202), octanoic acid (C8:0, m/z = 216), nonanoic acid (C9:0, m/z = 230), decanoic acid (C10:0, m/z = 244), dodecanoic acid (C12:0, m/z = 272), tetradecenoic acid (C14:1, m/z = 298), tetradecanoic acid (C14:0, m/z = 300), pentadecanoic acid (C15:0, m/z = 314), hexadecenoic acid (C16:1, m/z = 326), hexadecanoic acid (C16:0, m/z = 328), heptadecenoic acid (C17:1, m/z = 340), heptadecanoic acid (C17:0, m/z = 342), octadecatrienoic acid (C18:3, m/z = 350), octadecadienic acid (C18:2, m/z = 352), octadecenoic acid (C18:1, m/z = 354), octadecanoic acid (C18:0, m/z = 356), eicosatetraenoic acid (C20:4, m/z = 376), eicosapentaenoic acid (C20:5, m/z = 374), eicosanoic acid (C20:0, m/z = 384).

**Fig 6:**
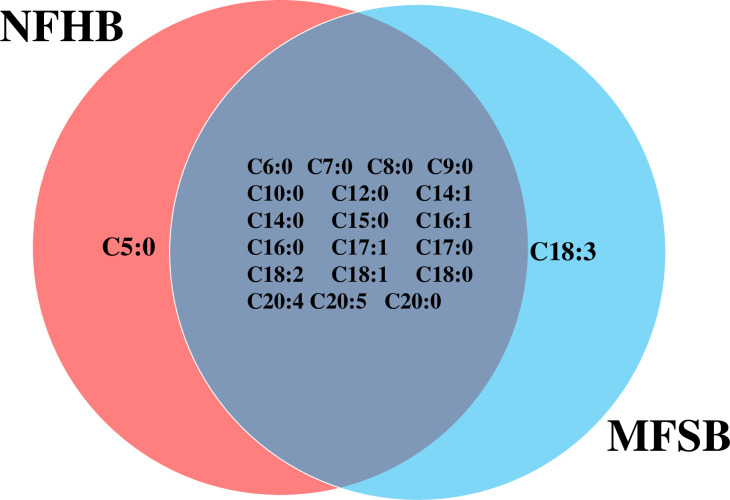
Venn diagram illustrating the differences in internal FFA in NFHB and MFSB mosquito.

Twenty FFAs were also observed in both the cuticular and internal NFHB extracts, but unlike the previous group, C18:3 was absent from both. The total ion current (TIC) chromatograms of fatty acids (TMS esters) of the dichloromethane extract after sonification (Extract III) from MFSB (A) and NFHB (B) has been shown in Fig 5. The difference in internal FFAs in each group is shown in the Venn diagram in Fig 6.

The dominant cuticular and internal FFAs were C16:1 (from 2.03±0.03 to 6.26±0.14 mg/g of insect body), C16:0 (from 1.23±0.01 to 4.32±0.07 mg/g of insect body), C18:2 (from 0.41±0.02 to 1.82±0.04 mg/g of insect body), C18:1 (from 2.86±0.14 to 7.47±0.24 mg/g of insect body), and C18:0 (from 0.26±0.01 to 0.90±0.03 mg/g of insect body). The lowest concentrations of cuticular and internal FFAs were found for C5:0 (from 0.89±0.16 to 2.86±0.51 μg/g of insect body), C7:0 (from 1.61±0.25 to 4.91±0.44 μg/g of insect body) and C10:0 (from 1.85±0.27 to 4.68±0.47 μg/g of insect body).

The cuticular extracts from the MFSB group demonstrated a higher concentration of all FFAs than the NFHB group, with the coefficient ranging from 1.18 (C5:0) to 5.59 (C17:1). In addition, the MFSB group also demonstrated higher FFA concentrations in the internal fractions of (except for C20:5) with the coefficient ranging from 1.2 (C14:1) to 2.02 (C17:1).

In the case of the NFHB group, higher concentrations of FFAs were mostly observed in the internal extracts than the cuticle extracts, with the exception of C5:0 (0.89±0.16 μg/g of insect body in internal extract versus 2.04±0.22 μg/g of insect body in cuticular extracts) and C6:0 (12.94±1.00 μg/g of insect body in internal extract versus 16.68±0.15 μg/g of insect body in cuticular extracts); in addition, the concentrations of C7:0, C9:0, C10:0 and C15:0 were also equal between the cuticular and internal fractions. In contrast, in the MFSB group, higher concentrations of all FFAs were observed in the cuticular extract with the exception for C18:3.

All extracts were found to contain glycerol. However, it was found at higher levels in the MFSB group (17.69±0.37 μg/g of insect body in cuticular and 29.20±1.97 μg/g of insect body in internal extract) than the NFHB group (5.39±0.44 μg/g of insect body in cuticular and 11.08±0.56 μg/g of insect body in internal extract). In both insect groups, higher glycerol concentrations were found in the internal extracts.

## Discussion

The membrane method is a popular means of providing blood to mosquitoes in laboratory conditions [51]. Its supporters emphasise the fact that mosquitoes fed using membrane feeders demonstrate similar female fertility and fed proportion (i.e. the proportion of females successfully fed) to those fed on live hosts [28, 29, 52–54]. Nevertheless, as feeder designs and membrane materials have a significant influence on the efficiency of feeding [24, 55, 56], they are continuing subjects of research. Our present findings indicate that the membrane feeding mosquitoes are characterised by 1.5- times lower body mass (per insect). In the present work, the mosquitoes fed by membrane feeders with sheep blood needed more time to fill their abdomens with blood (about one hour) than those fed on human blood (10 minutes). Similarly, Deng *et al*. reported lower fed proportions among female mosquitoes fed by membrane feeders [57]. Previous studies suggest that such differences in feed rate may result from a number of factors, including heat, odour, CO_2_, visual cues, blood pressure or membrane hardness [58–60]. The fact that the first five of these factors are present in an animal model but not in a membrane feeder could account for the preference of the mosquitoes for the former. However, Deng *et al*. reported that differences in feed rates were not present among third generation female mosquitoes, suggesting that they could become accustomed to membrane feeders; they may lose the ability to recognize the attraction factors of living skin and hence the potential to feed on live human hosts [57].

Female yellow fever mosquitoes might take several blood meals during each egg-laying cycle, so the supplies of blood in laboratory conditions is crucial [61]. Scott and Takken highlighted the unique adaptations of *Ae*. *aegypti* mosquitoes that allow them to live in association with humans [62]. As a result of habitat preference *Ae*. *aegypti* might prefer human blood than blood from other species [24, 62, 63], perhaps due to differences in the composition of human and animal blood. Harrington *et al*. proposed that their feeding preference may be associated with a low concentration of the amino acid isoleucine in human blood [19]; which has been found to play a role in the accumulation of mosquito energy reserves, survival and egg development [64]. A number of studies have reported lower fertility among yellow fever mosquitoes fed on sheep blood [28, 29, 65]. However, due to its low cost and wide availability, sheep blood is commonly used in laboratory conditions.

The present work examined the differences in the cuticular and internal FFA profiles between mosquitoes reared on sheep blood via membrane (MFSB) and those naturally fed on human blood (NFHB). One observation clearly demonstrates that the method of feeding and type of blood affects the proportion of FFAs in mosquitoes: MFSB demonstrated significantly higher levels of FFAs in both the cuticular and internal extracts compared to NFHB; in addition, while greater FFA concentrations were observed in the cuticular fraction among MFSB, higher FFA levels were found in the internal fraction among NFHB. This difference may be related to the changes in egg production by the female mosquito, as the process of reproduction is highly dependent on lipids [45]. Large amounts of lipids, which main role is to supply energy to the developing embryo, have been shown in *Culex quinquefasciatus* eggs where it was shown that about 90% of energy is derived from lipids [66].

The disproportionate lipid distribution observed around the body of the MFSBs, and their high concentration of their cuticular FFAs, suggests greater investments in the cuticle than in the reproduction process, and a reduction in fertility. Ross and co-workers reported lower fertility among mosquitoes fed with membrane methods using collagen as a membrane in the feeder [58]. In addition, lower egg-laying and hatching rates of *Anopheles* species was described after sheep blood meal [28]. Although the lower fertility of females fed sheep blood may be associated with the progression of transport and accumulation of FFAs in the cuticle, but detailed further research is needed to establish the mechanism responsible for this process.

The cuticle is the first barrier of the insect body and an important factor determining resistance to pathogens and xenobiotics, including commercial insecticides. Two main parameters play a key role in its resistance: the thickness and composition of the cuticle [67]. Literature data confirms that increased mosquito cuticle thickness is associated with greater resistance to insecticide. For example, *Anopheles funestus* or *Anopheles gambiae* with thicker cuticle layers demonstrated greater resistance to pyrethroid; however, the authors also highlighted that hydrocarbon content is significantly greater (about 30%) in resistant individuals [68–70]. The main factors involved in the synthesis of hydrocarbons are the cytochrome P450 enzymes of the CYP4G subfamily and long-chain FFAs, which act as precursors [71, 72]. However, no literature data currently exists about the role of cuticular FFAs in mosquito resistance or sensitivity, and more research in this area is needed. Moreover, mosquitoes have also demonstrated increasing resistance to insecticide after blood meal; this was probably connected with the changes taking place in the regulation of detoxification enzymes to adjust to the metabolic challenge posed by blood ingestion [73–75].

Mosquitoes can be controlled using entomopathogenic organisms such as fungi [76–80]. Although the role of blood meal in resistance to entomopathogenic fungi is unknown amongst mosquitoes, Cabral and co-workers indicated that *Ae*. *aegypti* females fed by mouse blood demonstrate higher resistance to infection by *Metarhizium anisopliae* than those fed with sugar, suggesting that blood components play key roles in the mosquito immune system possibly thought the rapid induction of Toll and IMD signalling pathways [81]. Similarly, Paula and co-workers reported that individuals infected immediately after a blood meal were more resistant to entomopathogenic fungus than those infected 122 hours afterwards [82]. Both studies highlight the importance of blood meal in the resistance to fungal infection by *Ae*. *aegypti* mosquitoes.

Literature data suggest that cuticular FFAs play a pivotal role in resistance or susceptibility to fungal infection; for example, in the flies *Calliphora vicina*, *Lucilia sericata*, *Calliphora vomitoria* and *Sarcophaga carnaria* [39, 43, 83–86]. In addition, the chemical composition of cuticular FFAs may influence the susceptibility of cockroaches (*Blatella germanica* and *Blatta orientalis*) to infection by the fungus *M*. *anisopliae* [87] or *Conidiobolus coronatus* [88]. Similarly, cuticular FFAs of corn earworm larvae (*Heliothis zea*), inhibit the germination and growth of entomopathogenic fungus *Beauveria bassiana* [89].

Our present findings indicate that membrane female feeding mosquitoes with sheep’s blood results in a significant increase in the concentration of C17:1 (5.5 times), C18:2 (4.4 times), C20:0 (3.6 times) C12:0 and C16:0 (both 3.5 times), C18:0 (3.4 times), C9:0 and C14:0 (both 3.2 times), C16:1, C17:0 and C20:4 (each 3.1 time), C6:0 and C7:0 (both 3.0 times) comparing to feeding naturally with human blood. All these FFAs possess antibacterial and antifungal properties: C17:1 inhibits the growth and germination of the fungi *Phytophthora infestans* and *Idriella bolley* and shows antimicrobial activity against entomopathogenic fungi: *M*. *anisopliae*, *Paecilomyces fumosoroseus*, *Paecilomyces lilacinus*, *Lecanicillium lecanii*, *B*. *bassiana* (Dv-1/07), *B*. *bassiana* (Tve-N39) and *Candida albicans*; in addition, FFA C18:2 has fungistatic activity against the entomopathogenic fungus *Erynia delphacis* and inhibits the growth of Gram-positive bacteria [85, 86, 90–93]. Gołębiowski and co-workers noted a correlation between the cuticular concentration of the FFAs C16:0, C18:0, C18:1 and the susceptibility of *Galleria mellonella* larvae to fungal infection [39]. In addition, FFAs C7:0, C9:0, C12:0, C16:1, C16:0, C18:1, C18:0, C18:2, C20:4 and C20:0 inhibit the growth and/or germination of spores, as well as the process of sporulation of the fungus *C*. *coronatus*, while the addition of fatty acids C6:0, C7:0, C9:0, C12:0, C16:1, C18:2 and C20:0 decrease infection ability and/or toxicity of metabolites released into the incubation medium by the fungus [94].

This variation in internal and cuticular FFA profile is believed to derive from the insect diet. Kazek and co-workers reported that diet affects cuticular FFA profile in *G*. *mellonella* and its sensitivity and response to microbial pathogens [95], while Rosumek and co-workers demonstrated that diet influences on FFA content in two species of ant: *Formica fusca* and *Myrmica rubra* [96]. In the present study, the higher accumulation of lipids observed in mosquitoes fed sheep blood (MFSB) could be explained by differences in the lipid content of sheep and human blood. An early study from 1937 reported protein to lipid ratios of 2.9 among sheep red blood cells (RBCs) and 4.4 among human RBCs, indicating lower protein content and higher lipid content in sheep RBCs [97]. However, sheep erythrocytes are not efficient meals for *Ae*. *aegypti* and decrease oviposition [26]. In the sheep plasma, the FFAs C18:2, C18:0, C18:1, C18:3 and C16:0 predominate [98]. A study from 1955 found that human blood contains less total protein than sheep blood, but twice as much total FFAs, 3.3 times more cholesterol and 2.1 times more phospholipids [99].

In the present work, both groups examined had access to sugar (saccharose). It is well known that female *Ae*. *aegypti* can feed on both blood and sugar in nature, depending on their availability [100]; however, when they can choose, they prefer bloodmeal [101]. The disproportion in FFA concentration between the two examined groups of mosquitoes might also be explained by the membrane-fed mosquitoes consuming more sugar, because sugar can be converted to lipids [102]. The greater mass of lipid extract observed for the mosquitoes on the sugar diet is consistent with literature data, because blood is metabolized more slowly than sugar and the level of synthesized lipid is five times higher after a sugar meal than after a blood meal [103, 104]. In addition, higher concentrations of lipid have been reported in the fat body after a sugar meal than a blood meal [45, 105].

Certain other factors may also affect the FFA content, such as differences in the composition of human and sheep blood and feeding behaviour, e.g. the amounts of blood drunk and the duration of blood meal. Moreover, it is also possible that heparinized sheep blood might have a different effect on FFA profile than fresh unprocessed human blood. It is well known that heparin administration induces lipolysis and increases the level of plasma FFA in sheep [106]. Moreover, research has shown that using heparin tubes to collect blood from dairy cows could affect the concentration of non-esterified fatty acids in whole blood and plasma [107]. Also spontaneous hydrolysis of esterified fatty acids into FFA has been reported in plasma and serum samples obtained from humans and other mammals in heparin tubes [108]. However, more detailed research is needed to determine whether the use of heparinized blood influences the FFA profile of mosquitoes.

Although insufficient scientific data exists on the origin of cuticular FFAs, it is likely that they are produced by the fat body and oocytes. The biosynthesis of saturated palmitic (C16:0) and stearic acids (C18:0) and monounsaturated oleic acid (C18:1) seems to be widespread among insects, and correspondingly these fatty acids are the most abundant in their bodies [43, 109]. In the present work, high concentrations of C16:1, C16:0, C18:1 and C18:2 acids, and lower amounts of saturated C18:0 were observed in both the cuticular and internal extracts. High concentrations of C16:1, C16:0 and C18:1 were also observed in extracts of the whole bodies of female *Aedes sollicitans* and *Aedes taeniorhynchus* mosquitoes, as well as an increase in the concentration of FFAs after a sugar meal, which might confirm that sugar feeding plays a role in lipid metabolism [110]. High amounts of the FFAs C16:1, C18:0 and C18:1 were also observed in *in vitro* cultured cells of *Ae*. *aegypti* larvae supplemented with fetal calf serum [111].

Van Handel and Lum reported a lack of polyunsaturated fatty acids in mosquito extracts [110]. However, high concentrations of C18:2 have also been observed in internal extracts from the biting midges *Culicoides obsoletus*, *Culicoides lupicaris*, *Culicoides fascipennis*, and *Culicoides kibunensis* and the non-biting midge *Forcipomyia bipunctata* [112], as well as in the present study.

In the present study, the polyunsaturated fatty acid C18:3 was only observed in extracts from the MFSB group. It was also previously observed in extract from whole *Musca domestica* and *C*. *vomitoria* imago after sucrose feeding, which might suggest that its synthesis may be associated with a sugar diet [43, 113]. In addition, Dadd suggested that C18:3 is crucial for flight ability in *Culex pipens*; it has also been identified in *in vitro* cultured cells of *Ae*. *aegypti* [111, 114].

In the present work, higher concentrations of eicosapentaenoic acid (C20:5) were found in the internal extract from the NFHB group than from the MFSB group, this being the opposite trend to that observed for other internal FFAs. Internally, the acid is present in the fat body and is considered as a precursor of prostaglandin, leukotrienes and thromboxanes [115–118]. Those eicosanoids play integral roles in immune functions, and are believed to mediate phagocytosis, encapsulation, and melanization responses to invading pathogens in insects [119–123]. Literature data suggests that eicosanoids contribute to the production of anti-microbial peptides, immune priming, and anti-*Plasmodium* immunity in *Anopheles*, but also influence the development of *Plasmodium* [124–128]. In *S*. *carnaria*, it has been found that males possess 90 times lower levels of C20:5 than females; the authors of this study suggest that this FFA may play a role in vitellogenesis [84]. Meanwhile Kaczmarek and co-workers have shown the 3.4 times higher concentration of C20:5 in internal than in cuticular fraction [129]. However, Gladyshev *et al*. reported the same amounts of C20:5 in extracts from larval and adult blood-sucking mosquitoes, suggesting that its level is highly conserved in mosquito [130]. In addition, Kumaratilake and co-workers reported this FFA to have antimalarial activity, as well as a nontoxic effect against normal red blood cells and parasitized red blood cells [131]. Sushchik and co-workers reported the transfer of essential eicosapentaenoic and arachidonic acids from storage lipids in larvae to functional polar lipids in adults during metamorphosis in a mosquito population of *Anopheles messeae*, *Ochlerotatus caspius*, *Ochlerotatus flavescens*, *Ochlerotatus euedes*, *Ochlerotatus subdiversus*, *Ochlerotatus cataphylla*, and *Anopheles cinereus*, inhabiting a temperate steppe wetland [132].

Glycerol is also produced by insects. It is thought to serve a cryoprotective function against freeze damage [133, 134]; however, it is also a well-known protein stabilizer protecting from denaturation through preferential hydration [135]. Higher concentrations of glycerol were noted in the internal lipid extracts than the cuticular extracts in both groups of mosquitoes, i.e. those fed with human blood and those fed with sheep blood; this could result from the hydrolysis of ingested di- and triglycerides and phospholipids. Higher concentrations of internal glycerol have also been observed in three female fly species: *M*. *domestica*, *C*. *vicina* and *S*. *carnaria* [136]. However, during this process, FFAs are also released, which might result in higher concentrations of internal FFA. Interestingly, no such findings were observed in the present work; for example, higher concentrations of glycerol but lower levels of FFAs were observed in the internal extract from the MFSB group than the NFHB group. Research on *Aedes albopictus* and *Ae*. *aegypti* mosquitoes indicates that the accumulation of glycerol might relate to glucose accumulation, and that Hsp70 may play a regulatory role in this process [134]. Glycerol is synthesized from fat body glycogen, and the observation that glucose can be accumulated as a fat or glycogen might explain the fact that glycerol is present in higher concentrations in extracts from the membrane-fed mosquitoes [137]. In the cuticle, together with the cuticular lipids, glycerol is believed to prevent water loss from the insect.

It is worth highlighting the lack of cholesterol in all examined extracts. Cholesterol is vital for membrane stability and cellular signalling, and serves as the precursor to the production of steroid hormones involved in regulation of yolk synthesis and egg maturation in oogenesis [138–141]. Cholesterol is also an important factor regulating insect development, and allows their life cycle to be completed [142]. However, mosquitoes are unable to synthetize cholesterol and its concentration is hence dependent on a blood diet [143]. Cholesterol has been observed in six species of mosquito larvae, *viz*. *Ae*. *aegypti*, *A*. *albopictus*, *Aedes epactius*, *A*. *taeniorhynchus*, *Anopheles stephensi* and *C*. *quinquefasciatus*, fed with a diet of rabbit chow [144]. In addition, the availability of nutrition during the larval instar regulates body size, lipid reserves and oogenesis in *Ae*. *aegypti* adults [145]. A lack of cholesterol in female adult insects is believed to be associated with a high degree of sterol incorporation into the egg [146].

In summary, the type of blood used to feed *Ae*. *aegypti* females changes the lipid metabolism of mosquitoes and has a profound effect on the FFA profiles of the cuticle and the internal parts of the insect. These changes may interfere with the fertility of the insect and its response to entomopathogens, xenobiotics and insecticides. These differences should be carefully considered when using mosquitoes in laboratory testing.

## Materials and methods

### Insects

*Ae*. *aegypti* female mosquitoes were obtained from Bábolna Bio (Hungary) and reared in the insectary room in the National Institute of Public Health—National Institute of Hygiene (NIPH-NIH). Mosquitoes were kept in the laboratory rearing room at a temperature of 25°C and RH 80% and a 12h/12h photoperiod. They were kept in 30cmx30cmx30cm tulle cages supplied with water and solution of saccharose in small containers (10%) *ad libitum*. Female and male individuals were kept together at a proportion of 1:2 males:females.

The life cycle of *Ae*. *aegypti* proceeds through four stages: egg, larva, pupa and adult. Imago life expectancy was about a week for males, and about a month for females. The fertilized females laid eggs to the water level on a wet paper tube half immersed in a crystallizer half-filled with water. The mosquito larvae were kept in the same temperature, humidity and photoperiod conditions as the adults and were fed fresh food for aquarium fish. The larval stage lasted eight days and the pupal stage one or two days.

### Blood feeding

The population of 3-days old mosquitoes was divided into two groups. The first (MFSB—mosquitoes membrane fed sheep blood) was composed of insects fed with defibrinated sheep blood with the use of a modified membrane method described previously in the NIPH-NIH laboratory [51, 147]. The feeder was constructed from a container filled with defibrinated sheep blood and covered with a membrane made from pieces of pig intestine. No sheep and pigs were directly involved in experiments. Before feeding, the blood was heated to 37°C in a water bath and mixed with using a magnetic stirrer to eliminate sludge. Each tulle cage was fitted with one feeder.

The second group of insects (NFHB) were fed naturally, i.e. with human blood. Three female volunteers put their arms into the mosquito cage and allowed the females to feed. All were researchers from the NIPH-NIH laboratory, and in accordance with bioethical commission procedures, had given their signed written consent to participate in the study (Bioethical Commission from National Institute of Public Health—National Institute of Hygiene). All experiments were performed in accordance with relevant guidelines and regulations. None of the volunteers had been supplemented by B vitamins few days before feeding, and on the day of feeding did not use cosmetics containing oils that could act as a repellent to insects (e.g. clove, vanilla, geranium or lemongrass). The feeding procedure ended when the majority of female mosquitoes had completed feeding and their abdomens were filled with blood. Natural feeding took approximately ten minutes, while the feeding through membrane took about one hour.

Some of the insects in each group was used to for mosquito colony husbandry. The remainder were used to test the FFA composition: around 90 mosquitoes were pooled and frozen 24 hours after blood meal. The numbers of individuals pooled and used for experiments are presented in Table 1.

### Extraction of FFAs

The cuticle and internal lipid components were extracted from the adult female insects described above, and then separated and analysed by Gas Chromatography–Mass Spectrometry (GC-MS) using highpurity solvents (≥95%, Merck Millipore). Firstly, the mosquites were extracted in 20 ml of petroleum ether for five minutes (extract I) and then a in 20 ml of dichloromethane for another five minutes (extract II). These two extracts (I and II) contained the cuticular lipids. The use of petroleum ether minimizes the possible extraction of internal lipids, which are mostly FFAs and glycerides [148]. The third extract was obtained by sonification of mosquitoes in 20 ml of dichloromethane for one minute. This extract contained the internal lipids. The extracts were placed in glass flasks and then evaporated under nitrogen. The masses of insects and the extracts are presented in Table 1. The extractions were performed only once from the entire pool of insects collected.

### Derivatization method

To obtain trimethylsilyl esters (TMS) of FFAs the mixture of one mg of each sample and 10 μl 19-methylarachidic acid (1 mg/ml; Merck Millipore) were silylated with 100 μl of N,O-Bis(trimethylsilyl)trifluoroacetamide (BSTFA): chlorotrimethylsilane (TMCS) (99:1) (Merck Millipore) for one hour at 100°C. The TMS values of the fatty acids were then analysed by GC-MS. The method is based on literature data [88, 95, 129, 149].

### GC-MS analyses

The analyses were carried out on a GCMS-QP2010 with a mass detector (Shimadzu). As a carrier gas, helium was used at a column head pressure of 65.2 kPa, with a DB-5 MS column (Zebron): thickness 0.25 μm, length 30 m, diameter 0.25 μm. The column oven temperature cycle started from 80°C; this temperature was held for three minutes then ramped to 310°C at 4°C/min; the final temperature was then held for 10 minutes. The ion source temperature was 200°C and the interface temperature was 310°C. The injection mode was split, and the split ratio was 10. All compounds were identified based on the fragmentation patterns and mass-to-charge ions of the TMS derivatives and the NIST 11 library. The mass spectra of the trimethylsilyl esters of fatty acids showed the following ions: M+ (molecular ion), [M-15]+, and fragment ions at m/z 117, 129, 132, and 145. As an internal standard (IS), 19-methylarachidic acid (1 mg/ml; Merck Millipore) was used to quantify each of the target compounds in the analysed samples, based on the areas of the chromatogram peaks. The results were expressed as means±standard deviation of μg substance per g of wet mass of insect body. The chromatographic analysis was performed three times for each sample. Response factors of one were assumed for all constituents. The method is based on literature data [84, 88, 95, 129, 149, 150].

### Statistics

The results of the chromatographic analysis, i.e. the absolute amounts of FFAs, were subjected to statistical analysis. The normality of their distribution was checked using the Kolmogorov-Smirnov (K-S) test. As the distributions were normal, the Student’s t-test was used to compare them at significance levels of 95% (p<0.05). STATISTICA software (StatSoft Polska) was used for all statistical testing. Each test was performed separately.

## Supporting information

S1 Table “Raw data”—Fatty acid contents in the cuticular (sum of I and II extract) and internal lipids (III extract) of the female mosquito (μg/g of insect body±SD). FFA- free fatty acid; SD—standard deviation; Extract I- petroleum ether extract; Extract II- dichloromethane extracts, Extract III- dichloromethane extracts after sonication; ND–not detected; MFSB–membrane feeding sheep blood; NFHB–natural feeding human blood. “normality of distribution”–The normality of distribution of data. FFA- free fatty acid; ND–not detected; MFSB–membrane feeding sheep blood; NFHB–natural feeding human blood. “Student’s t-test”–Statistically significant differences observed between the two groups regarding the amounts of FFAs on their cuticles and internal. Extract I–petroleum ether extract; Extract II–dichloromethane extracts; Extract III–dichloromethane extracts after sonication; FFA–free fatty acid; obtained results were tested using the nonparametric Student’s t-test, ** p<0.001; *p<0.05.
(XLSX)
